# Life cycle assessment of soil carbon dynamics in peach and olive cultivation in Greece: Implementing nature-based solutions

**DOI:** 10.1371/journal.pone.0325757

**Published:** 2025-06-20

**Authors:** Daphne Kitsou, Paraskevi Chantzi, Georgios Galanis, Dimitris Gkoutzikostas, Vasilios Roussonikolos, Konstantinos Karyotis, Maria Giortsou, Nikoleta Tiliopoulou, Argiro Papastergiou, Georgios Zalidis

**Affiliations:** 1 InterBalkan Environment Centre, Lagadas, Greece; 2 Faculty of Agriculture, Forestry, and Natural Environment, Aristotle University of Thessaloniki, Thessaloniki, Greece; University of Kalyani, INDIA

## Abstract

Carbon sequestration is a natural process that removes carbon dioxide from the atmosphere and stores it in plant biomass, soils, oceans, and geological formations- a very important process for reducing greenhouse gas concentrations, mitigating climate change and improving soil health. This study explores the environmental impact of implementing Nature Based Solutions (NBSs) in peach orchards and olive groves in Greece, using a cradle-to-farm-gate Life Cycle Assessment (LCA) approach. It specifically examines the chemical, physical, and soil conditions necessary for humification and organic carbon accumulation. The objectives are to: (1) assess the effectiveness NBSs in enhancing soil carbon sequestration within different agricultural management practices, (2) quantify the impact of cultivation transitions on soil carbon storage capacity and greenhouse gas (GHG) emissions, (3) integrate soil carbon stock dynamics into LCA methodologies to improve accuracy in carbon footprint assessments, and (4) highlight the importance of soil organic carbon (SOC) pools and soil properties in refining LCA interpretations for sustainable land management. By Implementing NBSs at pilot sites and using the Monitoring, Reporting and Verification system to track GHG emissions and SOC changes in the field, carbon stocks increased: −179.2 kg CO₂ eq in the peach orchard and −186.3 kg CO₂ eq in the olive grove. GHG emissions reduced by 16.4% in peach cultivation and 51.1% per hectare in olive grove. Main emission sources included crop protection and field energy use; in olives, residue management also played a significant role. This study provides empirical evidence on how NBSs can enhance soil carbon sequestration and reduce GHG emissions, contributing to both climate change mitigation and sustainable agricultural management. These findings demonstrate the value of integrating SOC dynamics into LCA for more reliable carbon assessments, offering a more accurate representation of carbon sequestration potential. The study can support policymakers, farmers, and stakeholders in adopting strategies that maximize environmental benefits and promote soil health. More long- term research on the implementation of Nature Based Solutions is required to properly assess their impact across cultivations, soil types and pedoclimatic zones.

## 1 Introduction

Soil stores three times the amount of carbon present in the atmosphere and could potentially remove from the atmosphere between 0.79 and 1.54 Gt yr^−1^ of carbon if land uses and management practices increased C inputs and/or reduced C losses [1]. Small changes in carbon stocks can impact the atmosphere and climate significantly. Since the start of agriculture thousands of years ago, large amounts of soil carbon have been lost due to cultivation. However, adopting better agricultural management practices can help restore a part of the carbon that is lost, making soil carbon sequestration an important strategy for reducing GHG emissions [2]. To reduce the threat of SOC losses while finding methods to improve soil carbon sequestration, it is essential to comprehend how land use and management affect SOC stocks [2]. Moreover, SOC improves soil nutrient availability, cation exchange capacity, water retention capacity, soil aeration, soil aggregation and structure, soil microbial biomass and its activity, plant yield, and quality [3–7]. The preceding seven years have been the warmest on record, according to the World Meteorological Organization (WMO) State of the Global Climate in 2021 report, while in 2020, human activity caused the atmospheric concentration of carbon dioxide to reach values above 410 ppm [8]. Furthermore, the average global temperature in 2021 rose to approximately 1.11 (± 0.13) °C above the pre-industrial level [9]. Reducing agricultural emissions has the potential to significantly mitigate climate change, as agriculture is a major contributor to anthropogenic global warming. Typically, aggregated CO_2_-equivalent emission rates are reported in a vague manner that fails to accurately represent past or projected contributions to changes in global temperature. Therefore, the standard way of describing emission reduction scenarios using CO_2_-equivalency obscures the roles and responsibilities of diverse sectors emitting various greenhouse gases [10]. Soil is becoming an increasingly important component in reducing greenhouse gas emissions from agriculture due to soil carbon storage, especially in view of the environmental problems caused by global climate change. The core principle of soil carbon sequestration is the absorption of CO_2_ from the atmosphere to the soil (aided by plants), plant wastes, and other organic substances. Thus, the necessity of carbon farming programs to raise SOC stocks is highlighted worldwide, offering a feasible way of addressing climate change [11].

Concern over rising carbon dioxide emissions and the pressing need for sustainable agriculture is increasing, bringing attention to Europe’s environmental initiatives. Central to these initiatives is the European Climate Law, a forward-thinking framework aimed at steering the European Union toward achieving carbon neutrality by 2050. In line with their commitment under the Paris Agreement, EU countries have agreed to become the first climate-neutral economy and society by 2050. This law helps EU countries incorporate smart climate practices in areas like agriculture. Recently, Europe introduced a carbon tax, linking environmental care with economic aspects by showing the real cost of carbon emissions on the environment and encouraging industries, including agriculture, to adopt eco-friendly methods. Aligned with the Climate Change Act’s principles, the carbon tax offers financial incentives for the agricultural sector to innovate in sustainable practices [12–14]. The European Green Deal, a package of policy initiatives, aims to set the EU on the path to climate neutrality by 2050 through a holistic and cross-sectoral approach involving climate, environment, energy, transport, industry, agriculture, and sustainable finance. The European Council notes that the transition to climate neutrality will bring significant opportunities, such as potential for economic growth, new business models and markets, new jobs, and technological development [15].

This case study follows the Greenhouse Gas (GHG) Protocol for accounting GHG emissions while implementing the Monitoring, Reporting, and Verification (MRV) system approach [16]. Developed by the World Resources Institute (WRI) and the World Business Council for Sustainable Development (WBCSD), the GHG Protocol is a widely used tool for governments and businesses to understand, quantify, and manage greenhouse gas emissions. It provides comprehensive guidance for organizations to identify emission sources, measure, and report them, covering direct emissions from stationary combustion of owned or controlled sources (Scope 1), indirect emissions from the generation of purchased electricity, heat, or steam (Scope 2), and other indirect emissions from agricultural activities (Scope 3). This structured approach allows organizations to identify emission hotspots, set reduction targets, and track progress over time. Internationally recognized as the most credible and rigorous standard for greenhouse gas accounting and reporting, the GHG Protocol is fundamental in advancing climate action by providing a common language and methodology for measuring and managing GHG emissions. The protocol relies on ISO standards, particularly ISO 14040:2006, which establishes principles for conducting life cycle assessments and includes ISO 14041 (scopes, goals, and Life Cycle Inventory methods), ISO 14042 (Life Cycle impact assessment methods), and ISO 14043 (Life Cycle interpretation), ensuring a robust and systematic approach to emissions accounting and sustainability assessment [17].

This study investigates the effectiveness of NBSs-recognized as carbon farming management practices that mimic natural processes to achieve climate resilience, in enhancing soil carbon sequestration and reducing GHG in Mediterranean agroecosystems. By using LCA methodologies, it aims to provide a comprehensive evaluation of the environmental impact of orchard management practices, contributing to sustainable land use strategies and climate change mitigation.

## 2 Materials and methods

### 2.1 Study sites description

**Fig 1:**
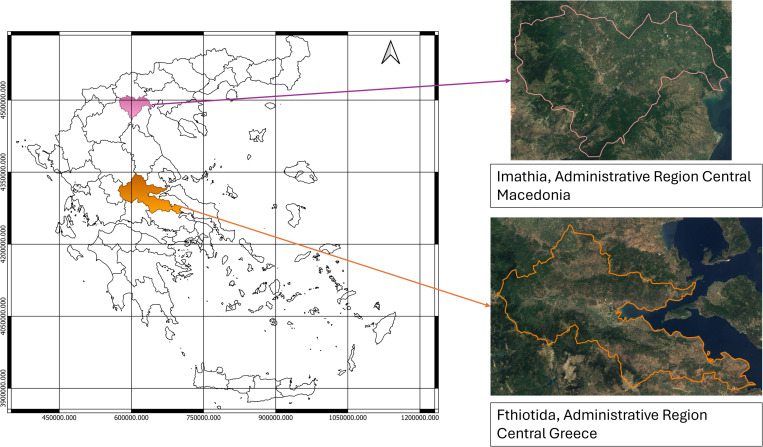
Map created in the QGIS environment using NUTS 2021 boundary data (Eurostat, CC BY 4.0) and Sentinel-2 Cloudless 2016 data - https://s2maps.eu by EOX IT Services GmbH (Contains modified Copernicus Sentinel data 2016 & 2017, CC BY 4.0).

This study was conducted in two different regions and pedoclimatic zones in Greece, one in Central Macedonia and one in the west-central part of the mainland (Fig 1). The first pilot site is located in the Imathia region, in Central Macedonia, named “Peaches”, a 9-year-old peach orchard consisting of 800 trees, size 1.2 ha. The area is formed by half lowlands and half highlands on the western side of the region, where the mountain Vermio rises. On the southern side, the valley of the river Aliakmonas is located, which serves as an important water supply for the area, among other small rivers. The climate is continental, with harsh winters and warm summers. The mean annual rainfall ranges between 400 and 600 mm on the lowlands and surpasses 1,200 mm on the highlands [18].

One pilot field located on the mainland of Central Greece was chosen from the Fthiotida region. The boundaries of the region are defined by the end of the Pindos Mountain range on the northwest side and Mount Timphristos on the southern side. The Vardousia mountain range is located in the southwestern area, whereas other smaller mountains are found east of Vardousians. The largest lowland in this region is around the drainage basin of the Sperhios River. The climate differs vastly across the region, with the highest rainfall occurring between October and May. The study was conducted for two consecutive cultivation periods, from October 2021 to September 2022, with the system boundaries cradle-to-farm-gate. The second pilot site is situated in Phthiotis and it’s named “Olives,” a 1.35 ha, 48-year-old olive grove, consisting of 180 trees [19].

### 2.2 Monitoring, reporting and verification (MRV) system approach and data collection

The European Green Deal [15], alongside its associated policies, aims to significantly reduce and eventually eliminate reliance on fossil carbon. Instead, it envisions sourcing carbon sustainably from ecosystems and industries, utilizing cutting-edge technologies. However, global efforts to combat climate change are falling short of achieving the CO_2_ atmospheric concentration goals set by the Paris Agreement [14]. Urgent scientific evidence underscores the need to actively reduce this concentration to mitigate global warming below 1.5°C. Enhancing methods for MRV in agriculture is a key component of the European Green Deal, crucial for understanding and mitigating GHG emissions, improving carbon storage, and reducing environmental impacts from farming activities. By adopting advanced monitoring tools like remote (i.e., satellite imagery) and proximal sensing (i.e., sensor networks), the EU aims to provide real-time insights into crop growth, soil conditions, and GHG emissions, enabling a detailed overview of farming operations and identifying areas with high emission levels and potential for carbon capture. Enhancing MRV capabilities not only yields more accurate emissions data but also supports informed policy decisions and encourages eco-friendly farming practices. Moreover, advancements in MRV techniques can promote performance-based incentive programs, rewarding farmers for sustainable practices, while technologies like blockchain enhance transparency, traceability, and data validity, ensuring credibility in reports of emission reductions. Beyond aiding climate change mitigation, robust MRV systems support sustainable land management and ecosystem conservation by guiding farmers toward practices that minimize harmful environmental impacts.

In light of these goals, implementing solutions for carbon removal through natural ecosystem methods like carbon storage is essential. However, strict MRV standards must be in place to ensure alignment with EU climate and environmental objectives. Considering factors such as storage duration and potential risks, transparent accounting for all forms of carbon removal is critical for achieving long-term climate goals.

Data collection and sampling were conducted with “The Field Diagnostic Toolbox”, an important part of the MRV system, which consists of a set of sensors that record field data (Earth In Situ data), soil indicators, and vegetation indices. This portable laboratory offers several advantages over laboratory facilities. It enables rapid and accurate measurements of volatile elements (e.g., nitrate in the soil) while allowing the collection of a large volume of cost-effective data. The Field Diagnostic Toolbox was developed by the Interbalkan Environmental Center- Green Innovation Hub to record Earth In Situ Data, which is necessary for the verification and debugging of Spaceborne Data. An integrated Earth Observation system with synchronized usage of Spaceborne and Earth In Situ can provide high-precision services to end-users based on their needs. The data are generated in the field using the toolbox sensors. The soil scanning system used is based on a spectral sensor employing microelectromechanical systems (MEMS) technology. This sensor records the diffuse reflectance spectrum in the near infrared (1,750–2,150 nm), which is affected by several key soil properties [20]. The data can be transmitted in real time (or asynchronously when internet connection is not established) to a centralized database through a mobile application. Data pass through a debugging and process unit, where a fusion process with ancillary and Remote Sensing data takes place. In addition to the soil spectrum, several metadata are recorded, including the timestamp, latitude and longitude of the sample, its humidity level, and more. The **indicators** supported by the Field Diagnostic Toolbox are presented in Table 1.

**Table 1:** Indicators “The Field Diagnostic Toolbox” produces. With * are the ones used in this study.

Indicator type	Specific indicators
Chemical Indicators*	Electric Conductivity, pH (1:1), N-NO3 (1:1), P-PO4 (1:1), K (1:1), CaCO3, Organic Matter, Soil Temperature
Physical Indicators*	Soil Texture, Soil Structure, Infiltration Rate, Soil Moisture
Biological Indicator*	Soil Basal Respiration
Plant Indicator	Normalized Difference Vegetation Index (NDVI)

Both pilot sites were sampled twice; once at the beginning of the cultivation period of 2021 and once at the end of the cultivation period of 2022, after the implementation of NBSs. The initial cultivation period at both pitot sites served as the baseline for this study. Sampling was limited to topsoil, 0–30 cm of disturbed and undisturbed soil samples. No special permits were required for taking samples from the field, since intervention was minimal without causing disturbances to the environment. Soil Organic Carbon (SOC) was validated in an ISO 17025 accredited laboratory using Walkey&Black method [21]. Additionally, the laboratory analysis included the measurement of soil texture.

### 2.3 Nature based solutions (NBSs) as agricultural management practices for carbon farming

Changes in SOC stocks significantly influence atmospheric carbon accumulation; thus, agricultural management practices aimed at increasing SOC stocks could have a massive impact on climate mitigation. Additional advantages include improved soil fertility, as increasing SOC stocks benefits both the physical and biological qualities of the soil. Agricultural intensification and land use change from grasslands to croplands have been linked to decreased SOC stocks. This decrease is intensified by agricultural practices with low organic material input, resulting in mechanisms such as oxidation/mineralization, leaching, and erosion [22]. Switching to restorative land uses and better agricultural management practices can improve soil quality and SOC. Carbon sequestration is closely related to soil management practices, especially those that preserve the soil.

Nature-based solutions refer to actions that mimic natural processes, and are used to address sustainably, land use, human well-being, and biodiversity. NBSs simulate natural processes and ecosystems in order to achieve climate change mitigation, adaptation, disaster risk reduction, water management, and biodiversity conservation. Utilizing NBSs as agricultural management practices can be an important pathway to climate change mitigation.

NBSs have been identified globally as very impactful to carbon sequestration. It has been demonstrated that the implementation of NBSs leads to carbon dioxide absorption and storage from the atmosphere with afforestation, reforestation, and sustainable land management practices. Furthermore, they are associated with biodiversity conservation and ecosystem resilience, furthering their importance in climate-change mitigation and adaptation [23].

The capacity of NBS to address a wide range of long-term challenges, including disaster risk mitigation, food safety, and climate change, has made them valuable instruments [24,25]. NBSs have achieved universal acceptance as a global tool for solving environmental concerns, and their adoption has grown significantly [25]. They are commonly used to restore vegetation, reduce soil degradation, and increase carbon sequestration [26]. These initiatives may preserve around 10 gigatons of CO_2_ equivalent annually by conserving ecosystems, regulating agricultural lands, and restoring degraded cover [27]. Notably, the Natural Climate Solutions World Atlas listed 15 particular NBS methods, such as reforestation, grazing management, and restoration of ecosystems [27,28].

Common agricultural management practices and Nature-Based Solutions for carbon farming that increase carbon sequestration are listed in Table 2.

**Table 2:** List of NBSs for carbon farming and carbon sequestration. With * are the NBSs used in this study.

List of nature based solutions for carbon farming and carbon sequestration			
Group of carbon farming practices	Carbon arming practice	Definitions	Sources
**Use of organic amendment**	Biochar	Carbon-rich material obtained by plant biomass pyrolysis	[29]
	Anaerobic digestate	Semi-liquid OA with fertilizer characteristics obtained from anaerobic digestion of plant biomass and/ or animal manure and slurry as by-product of biogas plants	[30]
	Compost	Humus-like material with fertilizer characteristics obtained from aerobic digestion of solid waste	[31]
	Farmyard manure	Decomposed animal feces mixed with stubble with fertilizer characteristics	[32]
Reduced soil disturbance*	Minimum tillage	Non-inversion tillage at maximum 15−10 cm depth	[33,34]
	No till	Sod-seeding	
	Reduced intensity tillage	Reduce number of tillage operation compared to business-as-usual	
	Reduced tillage	Non-inversion tillage at maximum 25 cm depth	
**Cover Crops**	Cover crops as green manure	Crops cultivated to obtain plant biomass are incorporated into soil with tillage operations	[35]
	Cover crops as green dead mulch	Crops cultivated to obtain plant biomass which is mowed/ trimmed and left on soil surface as dead mulch	
Agronomic management*	Intercropping	The practice of growing two or more crops in a field at the same time	[36]
	Improved crop rotations	Practice of growing different crops in recurrent succession on the same land	[35]
	Conservation agriculture	Agronomic management applying reduced soil disturbance combined with maintenance of crop residues, crop rotations, cover crops, inorganic fertilizer application)	[37]
	Organic agriculture	Organic farming is defined by the Reg. UE 2018/848	[38]
	Crop residues	Maintenance and incorporation of crop residues on field	[39]

### 2.4 Life cycle assessment (LCA)

Following a cradle-to-farm gate approach, Life Cycle Assessment (LCA) stops immediately after harvesting and before the product leaves the farm gate for storage or processing. The management practices and the inputs of the peach orchard and the olive grove are considered, setting the baseline right before the practices start. Other information used in the LCA are soil texture, soil organic matter (%), years passed from measurement, soil drainage, and soil pH. LCAs for net GHG emissions, for the total yield of peaches and olives, for each cultivation period, were conducted for the pilot sites “Peaches”, in Central Macedonia, and “Olives”, in Fthiotida.

LCA analysis was performed using the Cool Farm Tool, and the aim was to calculate the carbon sequestered after the implementation of the Nature-Based Solutions as agricultural management practices and to assess and compare the total Greenhouse Gases emitted per year. LCA was conducted twice for each pilot site, once before any management practices were implemented and once after the implementation of nature-based management practices after one cultivation period (incorporation of plant residues in the soil, in combination with no tillage). The LCA results were named “Peaches_2021” and “Peaches_2022”, and “Olives_2021” and “Olives_2022” according to the year in which the data were collected. Data sources included soil sampling and laboratory analyses. Field data were generated using the Field Diagnostic Toolbox and collected through crop logistics. Soil data were compiled and analyzed in the first year of the study, whereas data concerning inputs and management practices were gathered from the farmers for each year of the study.

Both types of tree crops are irrigated through drip irrigation systems. Electricity was used for irrigation (Greek energy mix, grid), and the fuel burned in agricultural machinery accounted for the entirety of the study. Plant biomass after pruning, fruit thinning, and grass chopping was integrated into the topsoil as a part of NBS management practice during the second cultivation period. The second NBS used during the second cultivation period was the no-till method (the previous method used was the conservation tillage). Emissions from agricultural practices, pesticides, fertilizers, and diesel burned in agricultural machinery are expressed in CO_2_ eq (kg). During this LCA, the following assumptions were made:

- The system boundaries for this study are considered cradle-to-farm gate.
- Data for the LCA were collected from the start of cultivation rather than during the complete life cycle. The LCA specifically excludes tree planting, orchard and grove establishment, nursery stages, soil preparation, and permanent infrastructure building (e.g., irrigation systems).
- LCA does not include the transit of main and secondary materials to the pilot sites (pesticides, fertilizers, plantlets, poles, etc.) as well as the production of the packaging used for such raw materials; instead, inputs in the system include the percentages of each active chemical compounds in fertilizers and pesticides applied to crops.
- The LCA for machinery does not cover the manufacturing, transportation, upkeep, replacement, and disposal of waste associated with machinery used in field activities. Diesel was the only fuel burned in agricultural machinery.

• The Life Cycle Assessment solely took account of the total amount (kg/ha) of agrochemical active substances. Additional compounds in agrochemicals were overlooked.
• LCA did not include human labor and transport of workers to the fields.
• The data used for the life cycle inventory (e.g., fuel consumption, used fertilizers, and irrigation) were retrieved from crop logistics provided by the farmers.

The Inventory is presented on Table 3.

**Table 3:** Inventory of inputs.

Scope	Input	Unit
Scope 1	Diesel	(MJ)
Scope 2	Electricity used for irrigation	(kWh)
Scope 3	Pesticides and insecticides	(Kg of active substance)
Scope 3	Fertilizers	(Kg of active substance)
Scope 3	Plant residues (only in the second cultivation period)	(Kg)
Scope 3	Yield	(Kg)
Scope 3	Land in occupation	(ha)
Scope 3	Water used for irrigation	(m^3^)

### 2.5 Calculations

Calculations made for this study include:

1. *Soil Organic Carbon,* C (%) = ((VT−VS) × 10 × 0,3 × 1,32)/ (WS×VT)
2. *where:*
3. *VS: volume of titrator used for the titration of the sample*
4. *VT: volume of titrator used for the titration of the blank sample*
5. *WS: mass of the sample used, measured in grams.*
6. *Organic Matter,* OM (%) = C (%) × 1.724
7. *where:*
8. *1.724 is the van Bemmelen factor [* 40 *]*

## 3 Results

### 3.1 Soil chemical properties

Soil chemical properties and their implications are important for determining carbon sequestration potential and nitrogen (N) uptake. This can provide helpful insights that contribute to a better understanding of the outcomes from the Life Cycle Assessment and draw conclusions regarding the use of Nature-Based Solutions as agricultural management practices. The soil chemical properties of the two pilot sites, “Peaches” and “Olives” are presented in Table 4.

**Table 4:** Soil chemical properties of the pilot sites. Sampling took place before the cultivation period of 2021.

Soil chemical properties	Peach orchard pilot site “peaches”	Olive grove pilot site “olives
*Sample Depth (cm)*	0-30	0-30
*Soil Texture*	50% Sand – 24% Silt - 26% Clay (SCL)	39.2% Sand – 18.1% Silt - 42.7% Clay (SCL)
*pH*	7.72	7.75
*Electrical Conductivity (EC) (mS/cm)*	0.486	0.462
*Organic Matter %*	0.85	0.92
*Calcium Carbonate (CaCO*_*3*_ *) %*	7.1	0.51
*Nitrate Nitrogen (NO*_*3*_ *) (PPM)*	10.29	12.18

### 3.2 Life cycle assessment

The results of the Life Cycle Assessments are presented below. Comparison charts were created between the baseline and the second year of each crop.

For the pilot site “Peaches” (peach orchard) in the year 2021, the majority of emissions came from electricity, irrigation, and crop protection. Management practices at the baseline left plant residues accumulated on the field and at a low level leading to no carbon sequestration and changes in carbon stocks. Crop protection accounted for 2,260 kg CO_2_ eq of the total emissions, while 32.5 kg CO_2_ eq were emitted from the use of fertilizers. Electricity used for the drip irrigation system accounted for 1,702 kg CO_2_ eq emissions, while the rest came from the diesel burned in agricultural machinery (1,088 kg CO_2_ eq). No changes in carbon stock due to management practices were observed. The conventional management practices implemented were low till and plant residues piled in a corner of the field, leading to a total of 736.7 kg CO_2_ eq emissions.

For the year 2022, the pilot site “Peaches” (peach orchard), the majority of emissions are attributed to energy consumption for irrigation and crop protection. Carbon stock changes were observed after the application of NBSs as management practices. Crop protection accounted for 2,170 kg CO_2_ eq of the total emissions, while 16.3 kg CO_2_ eq were emitted from the application of fertilizers. The total emissions from energy and fuel (diesel) amounted to 2,280 kg CO_2_ eq. The electricity used for the drip irrigation system accounted for 1,395 kg CO_2_ eq emissions, while diesel burned in agricultural machinery released 889.6 kg CO_2_ eq. After the implementation of management practices (NBSs: no-till, incorporation of plant residues) a total change of 179.2 kg CO_2_ eq was noticed.

**Fig 2:**
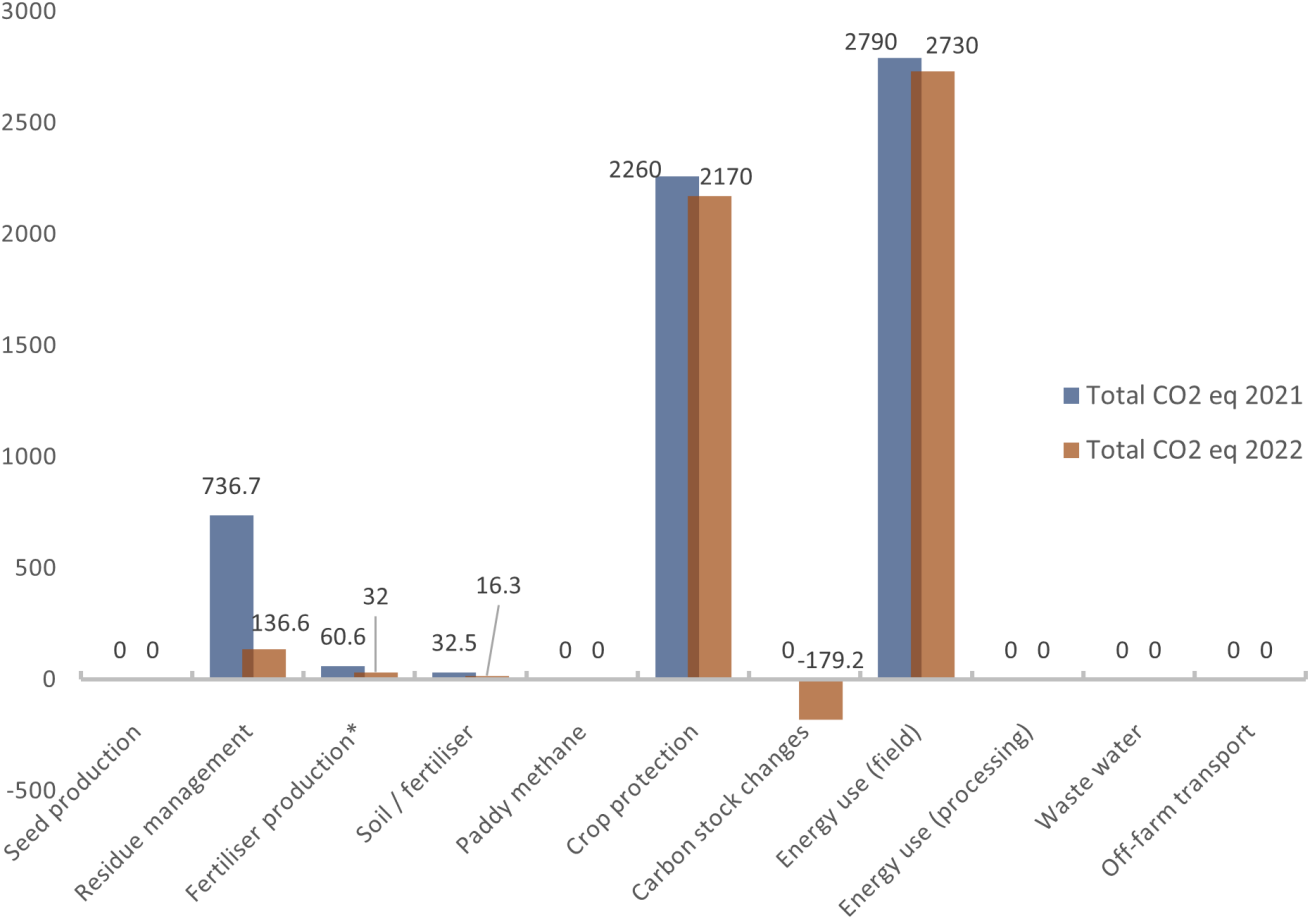
Comparison of the total emissions of Greenhouse Gases, expressed in kilograms of CO_2_ eq, for the pilot site “Peaches”, cultivation periods 2021 and 2022.

In the following figure (Fig 2) the comparison of the total emissions of Greenhouse Gases is presented. The total greenhouse gas emissions regarding the pilot site “Peaches” decreased from 5,870 kg CO_2_ eq in 2021–4,910 kg CO_2_ eq in 2022. Emissions per hectare decreased from 4,890 kg CO_2_ eq to 4,090 kg CO_2_ eq, and emissions per ton decreased from 101.3 kg CO_2_ eq to 84.6 kg CO_2_ eq. The GHGs are expressed in kilograms of CO_2_ eq, for the pilot site “Peaches”, for the cultivation periods of 2021 and 2022. As shown, total emissions from residue management, fertilizer emissions, agrochemicals, and energy are significantly lower in 2022, after the implementation of Nature-Based Solutions as agricultural management practices, resulting in soil organic carbon stock changes and potential carbon sequestration.

For the pilot site “Olives” (olive grove), for the cultivation period of 2021, the majority of emissions were attributed to the energy used for irrigation and plant residue management. Crop protection was responsible for the emission of 220.3 kg CO_2_ eq, while 63.3 kg CO_2_ eq were emitted from the use of custom soil fertilizers (N-P-K). The total emissions from the energy and fuel used in the field were 1,520 kg CO_2_ eq. Electricity used at the field accounted for 880.1 kg CO_2_ eq emissions; the drip irrigation system emitted 68.4 kg CO_2_ eq, while diesel burned in agricultural machinery released 304 kg CO_2_ eq. No carbon stock or sink changes owing to management practices were observed. The conventional management practices implemented were low till and plant residues were piled in a corner of the field, leading to a total of 14,490 kg CO_2_ eq emissions.

For the year 2022, the pilot site “Olives” (olive grove), during the cultivation period, most of emissions were attributed to energy used for irrigation, and plant residue management. Crop protection emissions summed up to 294.6 kg CO_2_ equivalent, comprising 96% of the total agrochemical emissions, and 11.7 kg CO_2_ equivalent were emitted from the use of fertilizers. The total emissions from the energy and fuel used in the field were 5,220 kg CO_2_ eq. Electricity used in the field accounted for 3,033 kg CO_2_ eq; the drip irrigation system emitted 104.4 kg CO_2_ eq, while diesel burned in agricultural machinery released 208.8 kg CO_2_ eq. After the implementation of management practices (NBSs: no-till, incorporation of plant residues) a total change in carbon stocks and sinks of 186.3 kg CO_2_ eq was noticed.

**Fig 3:**
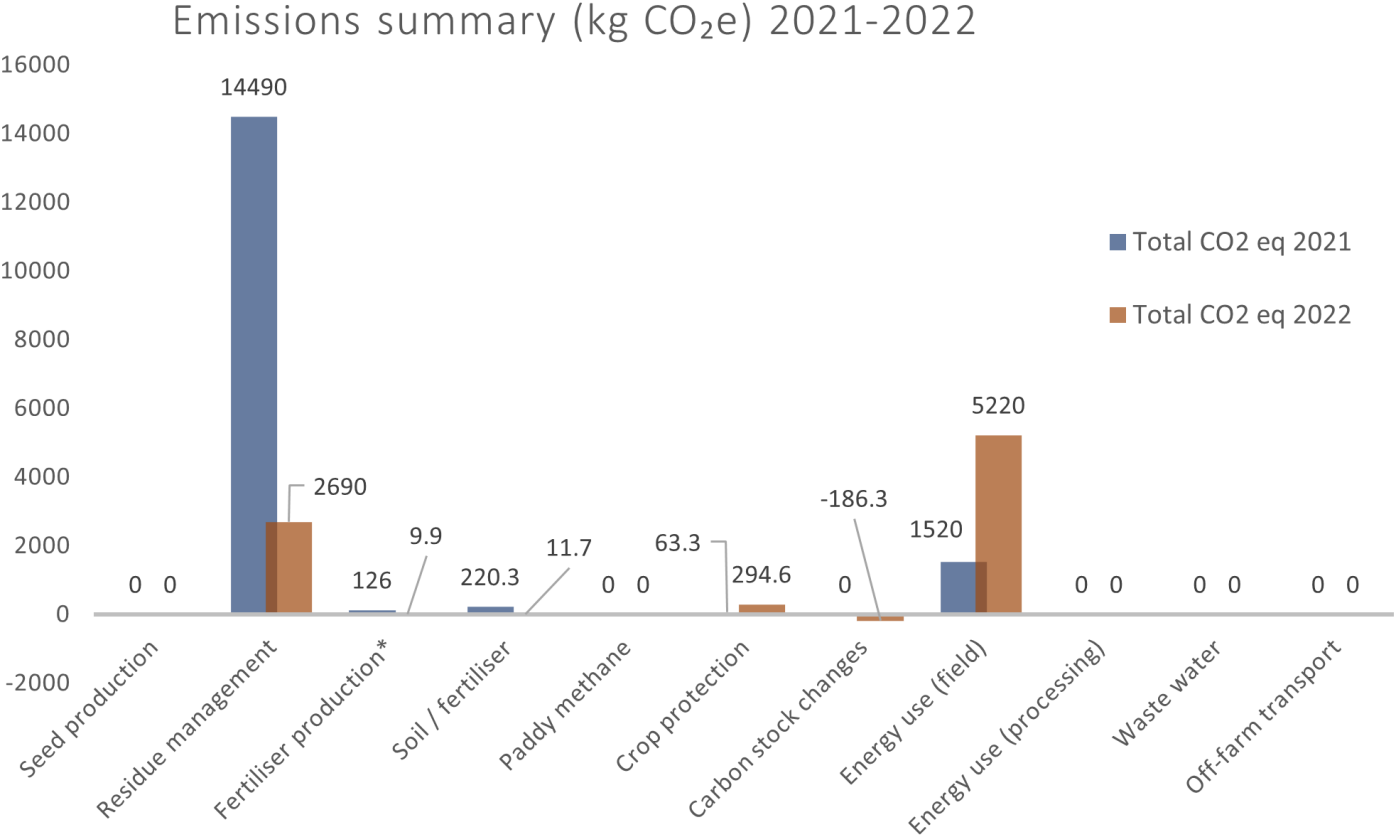
Comparison of the total emissions of Greenhouse Gases, expressed in kilograms of CO_2_ eq, for the pilot site “Olives”, cultivation periods 2021 and 2022.

In the following figure (Fig 3) a comparison of the total emissions of Greenhouse Gases is presented, expressed in kilograms of CO_2_ eq, for the pilot site “Olives”, for the cultivation periods 2021 and 2022.

As shown, the total emissions from residue management, fertilizer emissions, and crop protection exhibited significantly lower levels in the year 2022. However, emissions from the energy used in the field were considerably higher in 2022 than in 2021. After the implementation of Nature-Based Solutions as agricultural management practices, soil organic carbon stock changes were noticed, potentially leading to carbon sequestration.

### 3.3 Total comparison of the GHG emissions

The total greenhouse gas emissions from the pilot site “Peaches” decreased from 5,870 kg CO_2_ eq in 2021–4,910 kg CO_2_ eq in 2022, with emissions per hectare dropping from 4,890 kg CO_2_ eq to 4,090 kg CO_2_ eq, and per ton from 101.3 kg CO_2_ eq to 84.6 kg CO_2_ eq. The major sources of emissions related to crop protection (2,260 kg CO_2_ eq in 2021; 2,170 kg CO_2_ eq in 2022), field energy use (2,790 kg CO_2_ eq in 2021; 2,730 kg CO_2_ eq in 2022), and residue management, which decreased significantly from 736.7 kg CO_2_ eq in 2021 to 136.6 kg CO_2_ eq in 2022 owing to the incorporation of NBS crop residue. For the pilot site “Olives,” total emissions dropped substantially from 16,430 kg CO_2_ eq in 2021–8,040 kg CO_2_ eq in 2022, with emissions per hectare decreasing from 12,170 kg CO_2_ eq to 5,960 kg CO_2_ eq, and per kg from 63.2 kg CO_2_ eq to 0.6 kg CO_2_ eq. The highest emissions were from residue management, although it significantly reduced from 14,490 kg CO_2_ eq in 2021–2,690 kg CO_2_ eq in 2022, followed by crop protection (63.3 kg CO_2_ eq in 2021; 294.6 kg CO_2_ eq in 2022), and field energy use (1,520 kg CO_2_ eq in 2021; 5,220 kg CO_2_ eq in 2022). At both sites, the implementation of NBSs in 2022 led to changes in carbon stocks and sinks, resulting in carbon sequestration.

Residue management seems to be an important source of emissions for both sites, but it’s much higher for “Olives” compared to “Peaches” in both years. Crop protection contributes more to emissions for “Olives” in 2022, whereas it is significant for both sites in 2021. Energy usage in the field is substantial for both sites but is notably higher for “Olives” in 2022. Overall, although both sites have similar major sources, such as residue management and energy use in the field, there are differences in the magnitudes of these emissions. The pilot site “Olives” tends to have higher emissions in these categories compared to the pilot site “Peaches”, while the emissions from crop protection also becoming more pronounced for “Olives” in 2022.

## 4 Discussion

### 4.1 Carbon sequestration potential

In comparing the soil properties of the Peaches and Olives sites, several factors emerge that could influence carbon sequestration potential and nitrogen uptake. Despite similar pH levels, the peach pilot site exhibits a lower clay content (26%) relative to the olives pilot site (42.7%). This difference in soil texture could impact water retention, nutrient availability, and soil structure, potentially affecting carbon storage and plant growth. Additionally, while both sites have alkaline pH levels conducive to nutrient availability, the peach site possesses a substantially higher calcium carbonate content (7.1%) than the olive site (0.51%). Calcium carbonate levels can influence soil pH and microbial activity, further influencing carbon sequestration processes.

When considering organic matter content, the οlive grove pilot site demonstrates a slightly higher percentage (0.92%) than the peach orchard pilot site (0.85%). Organic matter is a crucial component for carbon sequestration, serving as a reservoir for carbon in the soil. Therefore, this disparity in organic matter content may have implications for the carbon sequestration potential of each site. Finally, the difference in nitrate nitrogen levels between the two sites (10.3 ppm for “Peaches” and 12.2 ppm for “Olives”) suggests variations in nitrogen availability, which could affect plant growth and productivity. Overall, these soil properties collectively contribute to the overall health and fertility of the soil, influencing carbon sequestration potential and nutrient cycling dynamics in these agricultural ecosystems.

Both sites reported a reduction in greenhouse gas emissions along with notable changes in emissions per hectare and per ton between the two years, indicating the effectiveness of implementing Nature-Based Solutions as agricultural management practices. The differences in greenhouse gas emissions between peach cultivation in Imathia and olive cultivation in Stylida can be attributed to several factors, including the unique needs of each crop, differences in climate and soil characteristics, and the influence of management practices and irrigation systems.

Peaches and olives have distinct growth characteristics and cultivation requirements. Peaches are deciduous fruit trees with relatively short growing seasons, whereas olive trees are evergreen and have longer growing seasons. Generally, the cultivation of peaches may involve more intensive management practices during the active growing season, including applications of fertilizers, pest and disease management (hence, higher crop protection emissions), and pruning activities. In contrast, olive cultivation may require less intensive management overall. Differences in crop management practices, such as pruning frequency or pest control methods, could influence the amount of greenhouse gas emissions associated with each crop.

Imathia and Stylida experience different climatic conditions, including variations in temperature, precipitation, and sunlight exposure. These climatic differences could affect crop growth, water requirements, and overall environmental conditions. The specific climate conditions of each region may influence factors such as irrigation needs, soil moisture levels, and the development of pests and diseases, which can in turn impact greenhouse gas emissions associated with crop cultivation.

The variance in electricity usage for irrigation between olives in Stylida and peaches in Veroia, despite olives requiring less irrigation, could be influenced by several factors:

*Water Pumping Efficiency:* The irrigation systems used for olives in Stylida may require more electricity due to factors such as higher lift heights, longer pumping distances, or less efficient pumping equipment. Even though olives exhibit lower overall water requirements, the efficiency of the irrigation systems can significantly impact electricity usage.
*Pressure Requirements:* The pressure requirements for drip irrigation systems can vary depending on factors such as elevation changes and the design of the irrigation system. If the irrigation system in the pilot site “Olives” demands more augmented pressure to maintain optimal irrigation distribution across the trees, it could lead to increased energy consumption compared to the pilot site in Veroia.
*Climatic Conditions:* While olive trees require less irrigation water, they may experience hotter and drier conditions compared to peaches in Veroia, due to different pedoclimatic zones. Higher temperatures occur in Stylida and can elevate evapotranspiration rates, leading to higher water demand during irrigation events. Consequently, even though olives require less water per irrigation cycle, the frequency of irrigation may be higher in Stylida, resulting in increased electricity usage.
*Soil Characteristics:* Differences in soil properties, such as texture and water-holding capacity, can influence irrigation requirements. The higher clay content and organic matter content in the soil of the olives site (Stylida) compared to the peaches site (Veroia) results in an enhanced water-holding capacity for olives.

While olives may require less frequent watering events due to the soil’s ability to retain moisture, the longer irrigation durations needed to replenish soil moisture levels in clay-rich soils can contribute to increased electricity usage.

Overall, while both sites employ similar management practices and irrigation systems, the differences in crop and soil characteristics, climate conditions, pedoclimatic zones, and local agricultural practices between Imathia and Stylida result in variations in greenhouse gas emissions associated with peach and olive cultivation. These factors highlight the importance of considering regional differences and specific crop requirements when assessing the environmental impacts of agricultural activities. Similar studies conducted in the Mediterranean region have reported positive results regarding enhancement of soil organic carbon stocks and GHG emissions reduction. Feudis et. al. [3] reported in their study, that took place in Italy, an increase of +82% on average in organic carbon stock after land use change and cover cropping from annual crops to orchards. Dichio et.al. [41] study in Italian orchards has shown that sustainable management practices (no-till, residue incorporation, cover crops, compost) in orchards can potentially mitigate climate change by 1.5 to 3.6 tons of carbon per ha/ per year, compared to conventional management practices. LCA analysis in this study returned a potential decrease of ~20% of GHG emissions with the use of sustainable management practices. Finally, Lal [42] has concluded through extensive research that the employment of sustainable management practices can result in carbon sequestration from 50 to 1000 kg per hectare per year. Lal estimates that using these methods might sequester 0.9 ± 0.3 Pg of carbon each year, potentially mitigating between a quarter and a third of the annual rise of atmospheric CO_2_ (estimated as 3.3 Pg of carbon per year).

## 5 Conclusions

Implementing NBSs as agricultural management practices to enhance carbon sequestration and mitigate GHG emissions is crucial for addressing climate change. No-till minimizes soil disturbance, helping conserve soil organic matter and maintain soil structure, which in turn improves aeration and reduces nitrous oxide (N₂O) emissions, which is produced during microbial processes. No-till also limits erosion, protecting carbon stored in soil aggregates and reducing sediment-related emissions to nearby water bodies. Returning crop residues to the soil after harvest increases carbon inputs, promotes long-term carbon storage in the soil, and enhances soil fertility, structure, and water retention. This practice also enhances biodiversity by providing habitat and food for soil microorganisms, essential for nutrient cycling and ecosystem functioning.

At both pilot sites, the application of these NBSs seemingly contributed to these objectives. Life Cycle Assessment (LCA) showed carbon stock increases of −179.2 kg CO₂ eq in the peach orchard and −186.3 kg CO₂ eq in the olive grove during the 2022 cultivation period. Given that the other inputs variables (crop protection, fertilizer application, diesel used in agricultural machinery, irrigation) remained relatively the same the carbon stock improvements, and potentially carbon sequestration in the stable carbon fraction, can be attributed to the implemented NBSs. However, further and more long-term analysis needs to be conducted regarding carbon fractions, pools, and sinks to ensure that the carbon stock enhancement that was achieved will not be oxidized in the immediate future (labile carbon fraction). However, site-specific pedoclimatic conditions played a significant role in the magnitude of sequestration observed. Stylida, where olives are cultivated, demonstrated higher carbon storage potential than Veroia (peaches), likely due to more favorable conditions—moderate temperatures, adequate moisture, and suitable soil types, supporting higher levels of soil organic matter accumulation and carbon storage. This highlights that even under identical management practices, environmental variables such as temperature, precipitation, soil composition, and topography can significantly affect the rates of carbon sequestration.The purpose of this study was to explore two primary research questions: initially, whether Nature-Based Solutions can be successfully used as agricultural management practices to improve soil health, biodiversity, and carbon sequestration; and subsequently, how the effectiveness of these solutions is impacted by different land uses, cultivation types, and specific pedoclimatic zones. By exploring these questions and their outcomes, the research covered a gap of case studies in Mediterranean highlighting the potential of Nature-Based Solutions in advancing sustainable agricultural practices while tackling environmental issues.

## Supporting information

S1 File Soil properties from pilot sites. (XLSX)
